# Annotations

**Published:** 1911-08-12

**Authors:** 


### ANNOTATIONS.

### Women in Camp at Tidworth.

Among the signs of the times that deserve record is the presence among troops of a woman's corps at the camp on Salisbury Plain. A party of twenty-three ladies belonging to the Winchester Division of the British Red Cross Society encamped last week at Tidworth. During their stay under canvas they succeeded in gaining a good deal of useful experience and in showing their capabilites for providing first aid assistance to the wounded. The possibility of allowing women to take an active part in military service in connection with ambulance work in the field is gradually being demonstrated, and the progress of this experimental movement will be watched with interest. As every statistician knows, the only reason why women have been prevented from engaging in active warfare from the earliest times has been that at least three times the number of women to men is required to keep up a community's birth-rate. Were two-thirds of the possible mothers in a nation killed, the excess of the male sex would be useless to replace them.

### The Claim of the Pit-brow Girls.

The sentimental feminists will not at first sympathise with the protest of the pit-brow workers against the clause in the Mines Bill which would prevent their following their work. The average masculine mind loves to think of woman as the light of the home, either as mother, daughter, or wife. How this light is to be provided in face of the need for domestic servants, factory-workers, and the like it does not trouble to inquire. But gentility rises against the spectacle of the girl at the top of the coal-pit, short-skirted, rough-booted, her head muffled in a kerchief, who is doing what is obviously rather dirty work. These girls are the sisters and daughters of pitmen; they are used to the sight of black faces, and they know that coal-dust is "clean dirt" which can easily be washed off at home. The factory girl may look less unpleasing to the eye when she quits her work, but when one considers the atmosphere in which she has been living, and the amount of cotton or other fluff which she inhales in the course of the day, one cannot wonder that local doctors—who, after all, do know more of the matter than the average M.P.—sometimes recommend girls to leave the factory and go to work at the pit-head. If the health of its women is a national asset, we should advise all working girls to go and pick coal or hoe turnips, or do any other outdoor work, rather than shut themselves up in the exhausted, hot, and humid air of a factory. If the visit of the pit-brow girls to the House of Commons serves no other purpose than to show M.P.s what healthy, hearty girls they are, it will not have taken place in vain, and may help to prevent legislation which, however well-intended, would tend to drive young women from healthy into unhealthy occupations. Anæmic, white-faced, round-shouldered girls are only too common among our women workers. Let us do nothing to drive into their ranks those who can earn their living by good, wholesome open-air work.

### Smoking and Cancer of the Tongue.

Both the British Dental Association and the International Dental Federation held an annual conference last week. The opening session of the latter was held at the Royal College of Surgeons where the President, Sir H. T. Butlin, occupied the chair and gave an interesting address. In mentioning how great was the importance of the condition of the teeth in relation to the general health of the community, Sir Henry Butlin expressed the hope that he would live to see the day when every person who practised odontology would be at the same time a fully qualified physician and surgeon. In the course of a lecture on leucoplakia and cancer of the tongue some interesting remarks were made on the relation of tobacco smoking to these affections. It is accepted that cancer on the tongue is very commonly associated with, and preceded by, leucoplakia, but the occurrence of the leucoplakia does not seem to bear any constant relation to indulgence in excessive smoking. People who smoke from morning till night often have healthy mouths, and on the other hand some quite moderate smokers have a white patch on that part of the tongue where smoke has constantly impinged. Irritation caused by a jagged tooth or by some condition of a stopping is undoubtedly an important factor which has to be reckoned with.

### Debased Ornamentation.

An Oxford graduate in arts and medicine has written all the way from the Central Provinces of India to criticise the following statement: "The chief factors, then, in the decline of the birth-rate are mechanical and logical. The mechanical factors just named are in reality only methods. It is the following logical factors that are the ultimate cause." The grounds of his quarrel are the sense of these remarks, not so much their inept, turgid expression; although the reflection that such a clumsy writer can hardly be a very clear thinker might have saved the trouble and ocean-postage expended. The point the gentleman quoted probably wished to make was the distinction between the how and the why, between the means by which a thing is effected and the reasons for doing it. Thus simply stated, however, to a certain taste the proposition seems too unimpressive and plain, and so some sounding but faulty verbiage is introduced to dress it up a little, also, perhaps, to fill up space—reminding one of the dictionary definition of the word *rococo*, which is a debased variety of ornament characterised by meaningless scrolls. It is strange that it is not commoner for medical men to imitate fervently the prose of Swift, Bunyan, and Cowper in what they have to write, especially as in many an ordinary spoken "clinical lecture" is to be found a shining example of the advantages to medical exposition of idiomatic terseness.

